# Engineered Protein Model of the ATP synthase H^+^- Channel Shows No Salt Bridge at the Rotor-Stator Interface

**DOI:** 10.1038/s41598-018-29693-z

**Published:** 2018-07-27

**Authors:** Hannah E. Pierson, Mandeep Kaler, Christopher O’Grady, Eva-Maria E. Uhlemann, Oleg Y. Dmitriev

**Affiliations:** 1 Department of Biochemistry, University of Saskatchewan, Saskatoon, Saskatchewan Canada; 2 Present Address: Department of Physiology, Johns Hopkins University, School of Medicine, Baltimore, MD USA

## Abstract

ATP synthase is powered by the flow of protons through the molecular turbine composed of two α-helical integral membrane proteins, subunit *a*, which makes a stator, and a cylindrical rotor assembly made of multiple copies of subunit *c*. Transient protonation of a universally conserved carboxylate on subunit *c* (D61 in *E. coli*) gated by the electrostatic interaction with arginine on subunit *a* (R210 in *E. coli*) is believed to be a crucial step in proton transfer across the membrane. We used a fusion protein consisting of subunit *a* and the adjacent helices of subunit *c* to test by NMR spectroscopy if *c*D61 and *a*R210 are involved in an electrostatic interaction with each other, and found no evidence of such interaction. We have also determined that R140 does not form a salt bridge with either D44 or D124 as was suggested previously by mutation analysis. Our results demonstrate the potential of using arginines as NMR reporter groups for structural and functional studies of challenging membrane proteins.

## Introduction

Integral membrane proteins remain a challenging target for structural biology. Among more than 100,000 entries in PDB, there are only about 700 unique structures of membrane proteins. Therefore methods that probe the local structure remain important, in addition to X-ray crystallography and cryoelectron microscopy, which can yield high-resolution structures of the entire membrane proteins. Among these methods, solution NMR is particularly informative due to its ability to resolve signals of chemically distinct atoms in the protein. Complete protein structure determination by NMR requires resonance assignment of most hydrogen, carbon and nitrogen nuclei in the protein molecule. Although several structures of polytopic α-helical membrane proteins, such as disulfide oxidoreductase DsbB^1^ and sensory rhodopsin^2^, have been solved by NMR, in general, such proteins are very difficult targets for NMR structure determination in solution due to the detrimental effects of fast relaxation in detergent micelles and chemical shift degeneracy. Fortunately, NMR spectroscopy can provide useful, if limited, structural information, even in the situations where only few resonances can be assigned. In the present work, we used NMR to probe the structural and functional role of arginine residues in subunit *a* of ATP synthase.

In the cell, ATP synthase makes ATP from ADP and inorganic phosphate. This process is driven by the proton motive or sodium motive force. In *E. coli*, the reaction is physiologically reversible and can generate transmembrane proton gradient using the energy of ATP hydrolysis. ATP synthase is composed of the F_1_-complex protruding into the cytoplasm, and the F_0_-complex immersed into the cell membrane^3^. ATP synthesis or hydrolysis in the active sites located in the F_1_-complex is coupled to the ion flow through the F_0_-complex.

In *E. coli*, the F_0_-complex is built of subunits *a*, *b*, and *c* combined in the ratio of *ab*_2_*c*_10_^4^. ATP synthase represents an ion-driven molecular turbine. Protons flowing through the F_0_-complex drive the rotor built of multiple copies of subunit *c*^5^, and connected to an elongated shaft made of F_1_-subunits γ and ε. The tip of subunit γ rotates inside the F_1_ core made of three pairs of subunits α and β, causing sequential conformational changes in the three catalytic sites on the β-subunits. Conformational transitions in the catalytic sites lead to the tight binding of ADP and phosphate and then to the release of the newly synthesized ATP molecules through the binding change mechanism^6,7^.

**Figure 1:**
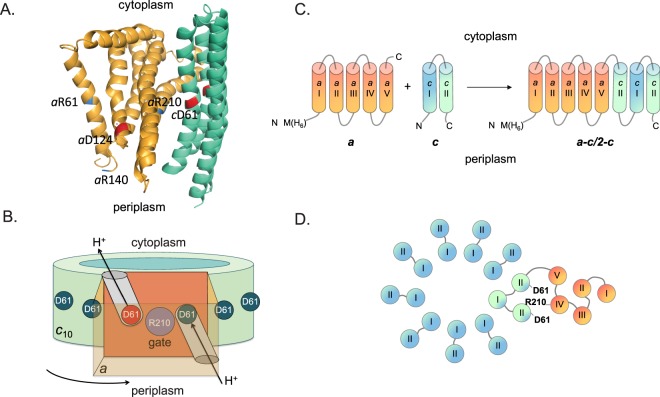
Structure of the proton channel subunits of *E. coli* ATP synthase. (**A**) Fragment of *E. coli* ATP synthase structure (PDB ID 5T4P) including subunit *a* (*orange*) and the two adjacent copies of subunit *c* (*green*). The arginine residues in subunit *a* are in blue (R24 is not present in the structure), and the aspartate residues in subunits *a* and *c* discussed in the text are in red (*a*D22 is absent from the structure). (**B**) Model of proton translocation in the membrane component of ATP synthase. Subunit *a (orange)* with the gating residue R210, and the *c-*ring (*c*_*10*_, *green*) with D61 on each *c* subunit are shown. Proton binds to the D61 carboxyl group of the *c-*subunit arriving at the exit of the periplasmic half-channel as the rotor turns in the direction shown by the arrow. Following an almost full turn of the rotor, this D61 releases the proton into the cytoplasmic half-channel. (**C**) Design of the fusion protein incorporating subunits *a* and *c* of ATP synthase (*a-c/2-c*). (**D**) Incorporation of the *a-c/2-c* protein into the F_0_-complex. The *c* subunits are in blue, the *a-c/2-c* protein is in green and orange, with transmembrane helices numbered with Roman numerals as for individual *a* and *c* subunits.

The high-resolution X-ray structures of the membrane component of ATP synthase rotor from various organisms^8–11^ show two concentric rings of α-helices, built of multiple hairpin-shaped *c* subunits, with the N-terminal helices (helix I) located in the inner ring, and the C-terminal helices (helix II) in the outer ring. Several recent structures of the whole ATP synthase^12–15^, mostly obtained by cryoelectron microscopy, completed the view of the F_0_-complex architecture. The stator subunit *a* consists of several α-helices and has a very unusual structure: four of its five transmembrane helices run at a very sharp angle to the plane of the lipid bilayer (Fig. 1A).

The transmembrane proton path in ATP synthase lies at the interface of subunits *a* and *c*^16–18^. When the enzyme is generating ATP, protons enter through the half-channel leading from the periplasmic side of the membrane to the carboxyl group of an aspartate or glutamate residue located on helix II of subunit *c*, within the hydrophobic slab of the membrane. As the subunit *c* ring rotates around the normal to the plane of the membrane, this carboxyl group shuttles the proton, almost full circle, from the entry half-channel to the exit half-channel, which leads to the cytoplasm, in the case of bacterial cells, or to the mitochondrial matrix in eukaryotic cells. Deprotonation of the rotor carboxyl group (*c*D61 in *E. coli*) and proton release into the exit half-channel is believed to be facilitated by the electrostatic interaction with the positively charged arginine side chain on subunit *a* (*a*R210 in *E. coli*)^19–21^ (Fig. 1B). However, the exact role of the essential arginine on subunit *a* remains uncertain. It also remains unknown, if the sidechains of R210 and D61 in *E. coli* (and the corresponding residues in the other organisms) come sufficiently close to each other during the rotor movement for the electrostatic interaction, as postulated by this model.

We used a model protein consisting of subunits *a* and *c* linked together in the correct transmembrane topology^22^ to probe the relative positions of *a*R210 and *c*D61 by solution NMR. We also tested the previously proposed role of *a*R140 in stabilizing the structure of subunit *a*. Our work demonstrates the use of arginine side chains as reporter groups for NMR studies of the membrane proteins that are not amenable to complete structure determination by NMR.

## Results and Discussion

### Arginine side chains in subunit *a* as NMR reporter groups

To analyze the interactions of arginine residues in the proton channel of ATP synthase, we used detergent purified subunit *a* and the previously designed model protein, which consists of subunit *a* fused to the C-terminal α-helix (helix-II) of subunit *c*, followed by a complete copy of the second subunit *c* (Fig. 1C). Our previous work^22^ shows that this protein incorporates into the complete ATP synthase in the correct transmembrane topology, and the arrangement of the transmembrane helices of the fused subunits *a* and *c* reflects the structure of the *a-c* interface in the native F_0_ complex (Fig. 1D). Both subunit *a* and the *a-c/*2*-c* protein were successfully purified in LMPG (Supplementary Fig. S1), a lysophospholipid, previously shown to produce good quality spectra of membrane proteins^23^. Our screening of various detergents with purified subunit *a* confirmed that LMPG produces the best spectral quality.

**Figure 2:**
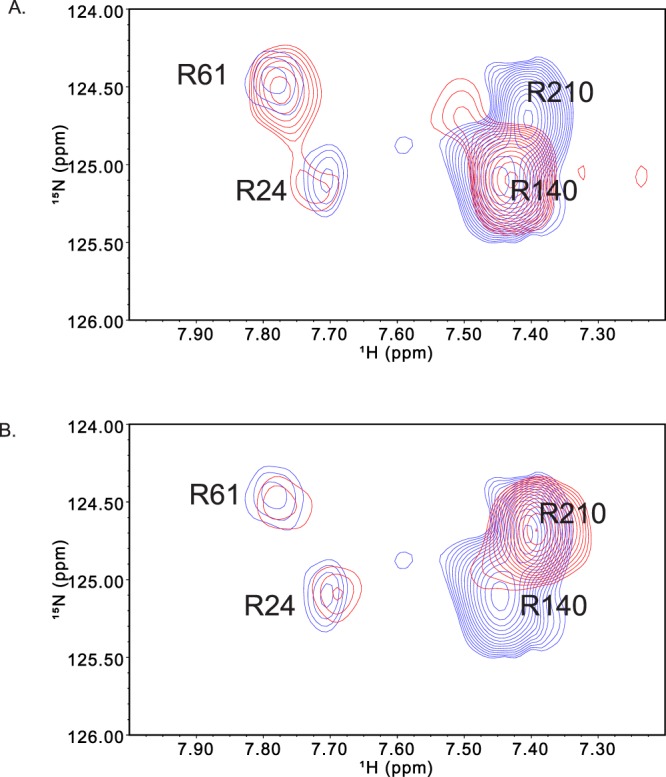
Arginine side chain region of the subunit *a* ^1^H, ^15^N- HSQC spectra. (**A**) Overlay of the spectra of the wild type subunit *a* (*blue*) and the R210Q (A) or R140Q (**B**) mutant variants. Signals of all four arginine side chain signals in subunit *a* are labeled. Note that the actual ^15^N chemical shifts of the arginine Nε are given by the formula δ = δ_obs_ − SW_N(ppm)_, where δ is the true chemical shift, δ_obs_ is the observed chemical shift, and SW_N(ppm)_ is the sweep width in the ^15^N dimension, expressed in p.p.m. (40 p.p.m. in our experiments). With the ^15^N frequency offset and sweep width commonly used in protein ^1^H,^15^N-HSQC experiments, arginine HNε signals appear in an uncrowded region of the spectrum and with an opposite sign to the backbone amides, allowing easy identification of the former.

Subunit *a* was found to be monodisperse in this detergent with the apparent molecular weight of the protein-detergent complex of 48 KDa, as estimated by size-exclusion chromatography. However, even under the optimal conditions, α-helical membrane proteins such as the 271 amino acid residue long subunit *a* and the 411 amino acid residue long *a-c/2-c* protein remain a serious technical challenge for NMR. Neither protein yielded sufficient spectral resolution to proceed with structure determination by NMR. However, the signals of the side chains of all four arginine residues in subunit *a* were well resolved and presented themselves as useful reporter groups to test several hypotheses regarding subunit *a* and the *a-c* interface structure. We were particularly interested in R210, the putative gateway residue in the proton channel of the ATP synthase, and in R140, which, on the basis of the earlier studies, was believed to form a stabilizing salt bridge with D124 of subunit *a*. We have assigned the signals of the R210 and R140 residues by comparing the spectra of the wild type and R210Q (Fig. 2A) or R140Q (Fig. 2B) subunit *a* variant respectively.

### Analysis of interactions of R140 and R24 in subunit *a*

**Figure 3:**
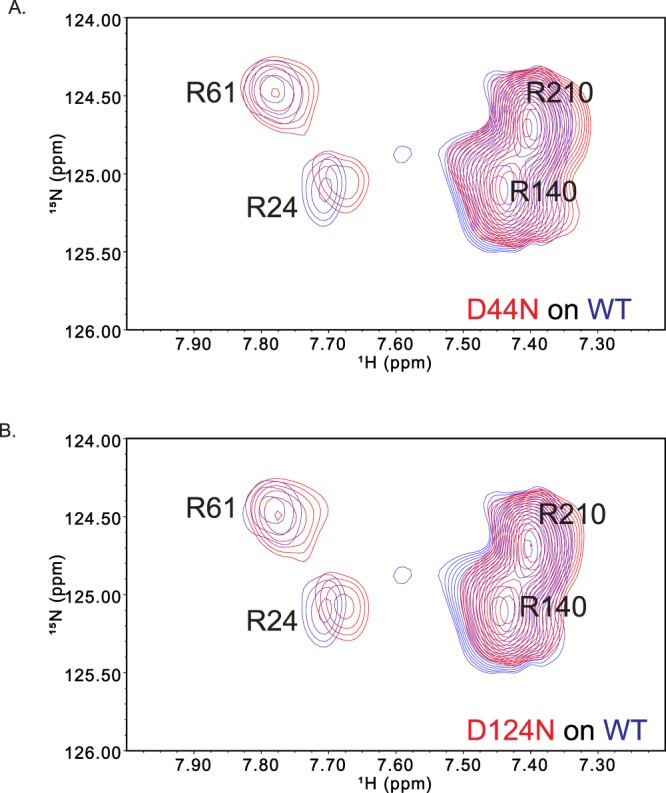
The chemical shift of R140 is unaffected by either D44N or D124N mutation. Overlays of the spectra of the wild type subunit *a* (WT, *blue*) and either D44N (*red*, A) or D124N (*red*, B) subunit *a* mutant.

We then used the assigned arginine signals to test several predictions about the subunit *a* structure. Based on the mutation analysis, residues R140 and D124 were proposed to stabilize the structure of subunit *a* by forming a salt bridge with each other^24^, with D44 possibly forming another salt bridge contributing to the stability of the R140-D124 one. The R140 is located in a loop region that is not well resolved in the EM structure of subunit *a*, and thus this structural feature still remained in question. Since chemical shifts are strongly influenced by the proximity of electrostatic charges, disruption of the putative salt bridge by mutating D124 or D44 to asparagine should change the chemical shifts of the guanidinium group in R140 side chain. However, neither D44N, nor D124N mutation had any significant effect on R140 chemical shift (Fig. 3).

**Figure 4:**
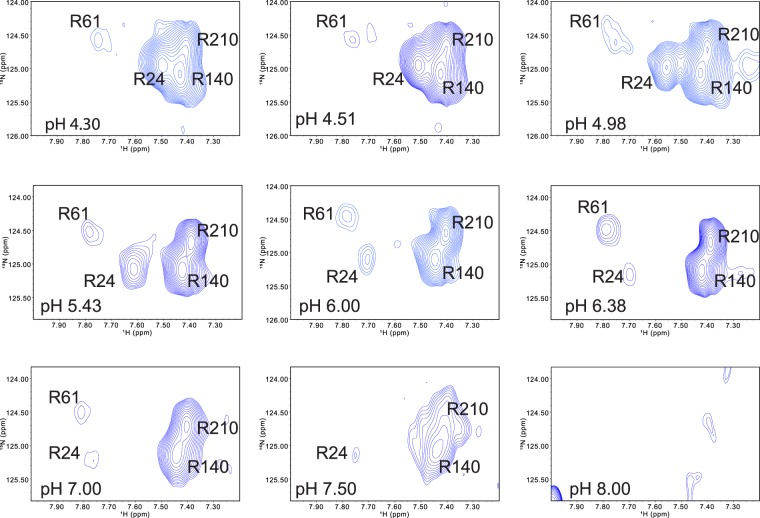
A pH-titration of subunit *a* monitored by the chemical shifts of the arginine side chains. Each spectrum was recorded at pH shown. Variable intensity of the signals is due to the pH-dependence of the proton exchange rates.

**Figure 5:**
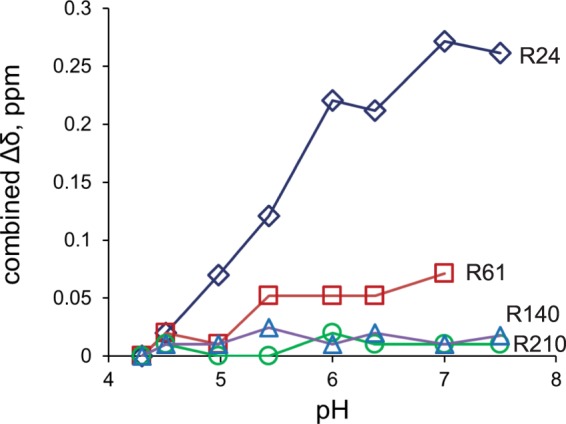
Combined chemical shift of HN_ε_ in subunit *a* arginines as a function of pH. Combined chemical shift change Δδ was calculated as [(Δδ^2^_HN_ + Δδ^2^_N_/25)/2]^1/2^, where Δδ_HN_ and Δδ_N_ are the chemical shifts changes of the proton and nitrogen respectively, relative to pH 4.3.

An alternative approach to test for the proximity of a carboxyl group to an arginine residue is pH-titration. Guanidinium groups in the arginine residues have high p*K*_*a*_ values, above 12.0, whereas carboxyl groups in the aspartate sidechains in proteins have p*K*_*a*_ = 3.5 ± 1.2, although values as low as 0.5 and as high as 9.2 have been reported^25,26^. Thus a change in the ionization state of an aspartate sidechain should influence the chemical shift of an interacting arginine. We conducted pH titration in the range pH 4.3–8.0, limited by the sample stability at the lower end and high proton chemical exchange rate at the upper end of the range. Neither R140, nor R210 sidechain signals showed any chemical shift pH-dependence in this range, but the signal of one of the other two arginine residues, either R24 or R61, experienced major secondary shift (Figs 4 and 5), consistent with titration of an ionizable group with p*K*_*a*_ of about 5.5 or below. There are no aspartate or glutamate residues in the proximity of R61, but there is an aspartate (D22) located just two residues away from R24. Titration of the D22 side chain is the likely cause of the observed changes in the arginine signal, which, on this basis, we provisionally assigned to R24. In summary, these results led us to conclude that R140 is not involved in a salt bridge with either D124, or any other residue in subunit *a*, and the disruptive effects of the D44 and D124 mutations must have a different explanation. The pH-dependent chemical shift R24 validates using pH-titration to detect potential electrostatic interactions involving arginine side chains.

### Arginine 210 of subunit *a* and Aspartate 61 of subunit *c* do not interact with each other

Using the pH-titration method, we addressed the question if *a*R210, the gateway residue of the ATP synthase proton channel, is located within the range of electrostatic interaction with *c*D61, the rotor residue, which is believed to shuttle protons between the two transmembrane half-channels. For this experiment, we used an engineered model protein that consists of subunit *a* fused to the transmembrane helix-II of subunit *c*, followed by another, complete *c* subunit (*a-c/2-c*, Fig. 1C).

In addition to the four arginine residues in subunit *a*, the *a-c/2-c* protein contains four arginines, R283, R292, R373 and R382 corresponding to R41 and R50 in the loop segments of the two *c* subunits. The signals of these arginines overlap with the signals of R210 and the other subunit *a* arginines (Supplementary Fig. S2A). Therefore we replaced them with lysines. These are conservative substitutions that are not expected to affect folding of the *a-c/2-c* protein. In fact, the R41K mutant was previously shown to have normal F_0_ proton conductance^27^. The positions of subunit *a* sidechain arginine signals remained unperturbed in the R283K/R292K/R373K/R382K quadruple mutant (Supplementary Fig. S2B).

**Figure 6:**
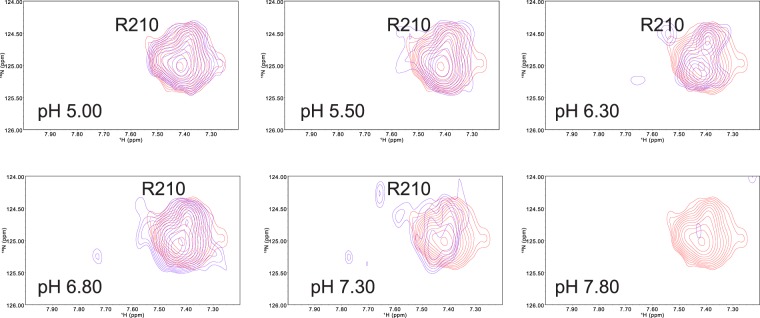
A pH-titration of the *a-c/2-c* protein monitored by the chemical shifts of the arginine side chains. Overlay of the spectra recorded at pH 4.5 (*red*) and at pH shown (*blue*).

Compared to the spectrum of subunit *a*, the chemical shifts of the R210 side chain did not change in the *a-c/2-c* protein (Supplementary Fig. S2B). This was the first indication that the aspartate residues D303 and D393 corresponding to the essential D61 residues in the two copies of subunit *c*, are not in the immediate proximity of the R210 sidechain in the *a-c/2-c* protein. We then conducted pH-titration by NMR in the range 4.5–7.8, and did not observe any secondary shift of R210 (Fig. 6). This is significant because the p*K*_*a*_ of *c*D61 was previously determined to be 7.1^28^, within the range covered by titration. This unusually high value likely reflects the hydrophobic environment of this residue. Taken together, these results indicate that in the *a-c/2-c* protein, the residues corresponding to D61 of subunit *c* and R210 of subunit *a* are not sufficiently close for a strong electrostatic interaction between the sidechains, certainly outside the distance range for a salt bridge formation between the side chains. This result is consistent with the cryo-EM structure of the F_0_ complex of the mitochondrial ATP synthase from yeast^29^, the first structure of the complete F_0_ complex that has sufficient resolution to accurately determine positions of the side chains of the essential residues on subunits *a* and *c*. In this structure, the R176 on subunit *a* and the nearest E59 on subunit *c*, corresponding to *a*R210 and *c*D61 in the *E. coli* ATP synthase, are still too far for salt bridge formation. This appears also to be true for *E. coli* ATP synthase^30^ (Fig. 1A), although the latter structure does not have sufficient resolution to visualize side chains of *a*R210 and *c*D61.

It should be noted that the model system employed in our study has certain limitations. Since the *a-c/2-c* protein, by design, covalently links the rotor and the stator components, it is not possible to validate correct folding of the protein by measuring ATP-driven H^+^-translocation in the membrane. Still, our previous work offers several pieces of evidence that the *a-c/2-c* protein correctly recapitulates the interface between subunit *a*, and the two copies of the *c* subunit in the native ATP synthase^22^. Although subunit *c* is required for incorporation of subunit *a* into the membrane, the L31F and G23D subunit *c* mutants, which do not assemble into the rings, support normal incorporation of subunit *a*, presumably through the formation of *ac* or *ac*_*2*_ complexes. Importantly, unlike subunit *a* by itself, the *a-c/2-c* protein incorporates into the lipid membrane in the absence of subunit *c*. Expressed in the context of the complete *atp* operon together with subunit *c*, the *a-c/2-c* protein supports normal assembly of ATP synthase, and the various transmembrane helices of the *a-c/2-c* protein correctly incorporate into the stator and into the rotor linking them together (Fig. 1D) and maintaining tight coupling between the F_0_ and F_1_ components of the rotor. In contrast, the *a-c* protein, which lacks one of the transmembrane helices derived from subunit *c*, and therefore has incorrect transmembrane topology, does not properly incorporate into the F_0_ complex^22^. Taken together, these results suggest that the native *a-c* interactions are preserved in the *a-c/2-c* protein, and that the assembled *c-*ring is not required for the proper folding of the stator-rotor interface region in ATP synthase.

Another concern, rather common in the structural studies of membrane proteins, is the possible effect of detergent on the *a-c/2-c* protein conformation. The *E. coli* F_0_-complex withstands purification in a rather harsh detergent without subunit dissociation, or loss of function^31^. Moreover, proton translocation is restored, when subunits *a, b*, and *c* are purified individually, following F_0_ dissociation with chaotropic agents^31^ or organic solvents^32,33^, and then recombined and reconstituted in liposomes. These observations suggest that the native *a-c* interactions in the F_0_ complex, and, by extension, in the *a-c/2-c* protein, are stable, not easily disrupted by detergents, and do not depend on the presence of specific lipids. To further reduce probability of protein denaturation, in our experiments we have used lysophosphatidyl glycerol (1-myristoyl-2-hydroxy-*sn*-glycero-3-[phospho-*rac*-(1-glycerol)], LMPG), which structurally closely resembles natural phospholipids. This detergent has been previously tested in the NMR studies of membrane proteins, including subunit *c* of ATP synthase^23^, and, recently, coronavirus E channel^34^, among others, and produced spectra indicative of structurally homogeneous, well folded proteins. Conceivably, solid state NMR in lipid environment could be used to further corroborate our results in the future.

In summary, the available data from different sources indicate that the essential arginine on the stator subunit *a*, and the rotor subunit *c* do not form a salt bridge. Although the proximity of these two residues and their universal conservation in ATP synthases seem to make the idea of salt bridge attractive, it would create a kinetic obstacle for the rotary mechanism of ion transport in ATP synthase. The low dielectric constant within the hydrophobic environment at the rotor-stator interface potentiates the electrostatic interactions, and the activation energy of breaking the salt bridge at each rotational step would be very high. If the role of the stator arginine is indeed to facilitate deprotonation of the proton-shuttling carboxyl on the rotor, a weaker electrostatic interaction at a longer distance between the two residues may be sufficient. Alternatively, the role of the stator arginine may be simply to prevent the backflow of protons up the exit channel by creating electrostatic repulsion between the positively charged arginine and the positively charged protons (or hydronium ions).

In conclusion, we have demonstrated how arginine NMR signals can be used to probe the structure and function of challenging membrane proteins. Using arginines in subunit *a* of *E. coli* ATP synthase as reporter groups, we tested the existence of two putative salt bridges in the F_0_-complex of ATP synthase, one that was proposed to stabilize the subunit *a* structure, and the other, which was proposed to be an essential part of proton transfer mechanism in ATP synthase. We did not detect either the *a*R140-D44/D124 or *a*R210-*c*D61 interaction. The latter finding suggests that no strong electrostatic interaction between the universally conserved stator arginine and rotor aspartate (or glutamate) residues is involved in proton transfer in the F_0_ complex of ATP synthase.

## Materials and Methods

### Protein expression and purification

The R140Q, D44N and D124N mutant variants of subunit *a* were generated by site-directed mutagenesis using the megaprimer method^35^. The fusion protein *a-c/2-c*, consisting of subunit *a*, transmembrane helix II of subunit *c*, and then a complete subunit *c* linked in a head-to-tail fashion (Fig. 1C), was further modified to prevent spectral overlap between the signals of the arginine residues in subunit *a*, and the arginines corresponding to R41 and R50 in the loop region of subunit *c*. The corresponding arginines were replaced with lysines, and an internal KpnI site was removed in a chemically synthesized *a-c/2-c* gene fragment, which was then cloned into the KpnI and NheI sites of the pHP808 plasmid^22^ replacing the corresponding fragment of the original *a-c/2-c* gene. All the protein variants included an N-terminal hexahistidine tag for affinity chromatography purification. Subunit *a* and the *a-c/2-c* protein were constitutively expressed in the context of the complete *atp* operon from plasmids pBWU13-*a*N-His_6_^33,36^ and pHP808^22^ respectively. *E. coli* strains C43(DE3)^37^ and C43(DE3)*recA*^−^ were used for expressing subunit *a* and the *a-c/2-c* protein respectively. The latter strain was generated by P1 transduction using BLR(DE3) (Novagen) strain as a donor.

### Protein purification and NMR experiments

Subunit *a* and the *a-c/2-c* protein were purified essentially as described previously^38^. Briefly, the *E. coli* cell cultures were grown on M9 minimal medium containing ^15^N-ammonium chloride (Cambridge Isotope Laboratories) as a sole source of nitrogen to the late exponential phase. Cells were disrupted using cell disruptor (Constant Systems Inc., Kennesaw, GA), and the cell membranes were isolated by ultracentrifugation^39^. The F_0_ complex was purified from the membrane fraction by the Schneider-Altendorf procedure^31^ and dissociated into individual subunits by LiBr treatment. Subunit *a* and the *a-c/2-c* protein were purified by Ni-NTA chromatography with on-the-column detergent exchange for 1-myristoyl-2-hydroxy-*sn*-glycero-3-[phospho-*rac*-(1-glycerol)] (LMPG), and equilibrated in the NMR sample buffer as described^38^. The standard NMR samples contained 0.2–0.4 mM protein in 10 mM KH_2_PO_4_, pH 6.0, 0.01% LMPG, with 5% (v/v) D_2_O and 0.3 mM 2,2-dimethyl-2-silapentane-5-sulfonic acid (DSS), added for frequency lock and chemical shift referencing respectively, in a total volume of 0.35–0.4 ml. The ^1^H, ^15^N-HSQC spectra were recorded at 45 °C on a 600 MHz or 900 MHz Bruker (Billerica, MA) NMR spectrometer equipped with a Cryoprobe and z-axis pulse field gradients. Water suppression was achieved using double pulsed field gradient spin echo^40^.

### Data availability statement

The datasets generated during and/or analyzed during the current study are available from the corresponding author on reasonable request.

## Electronic supplementary material

Supplementary information
