# Supplementary material for: Engineered Protein Model of the ATP synthase H+- Channel Shows No Salt Bridge at the Rotor-Stator Interface
Source: Sci Rep. 2018 Jul 27;8:11361. doi: 10.1038/s41598-018-29693-z (PMC6063947; doi:10.1038/s41598-018-29693-z)
Supplement: Supplementary file 1 — Supplementary information [file 41598_2018_29693_MOESM1_ESM.pdf]

Engineered Protein Model of the ATP synthase H^+- Channel Shows No Salt Bridge at the Rotor-Stator Interface

Hannah E. Pierson, Mandeep Kaler, Christopher O'Grady, Eva-Maria E. Uhlemann, and Oleg Y. Dmitriev

Supplementary information

Fig. S1

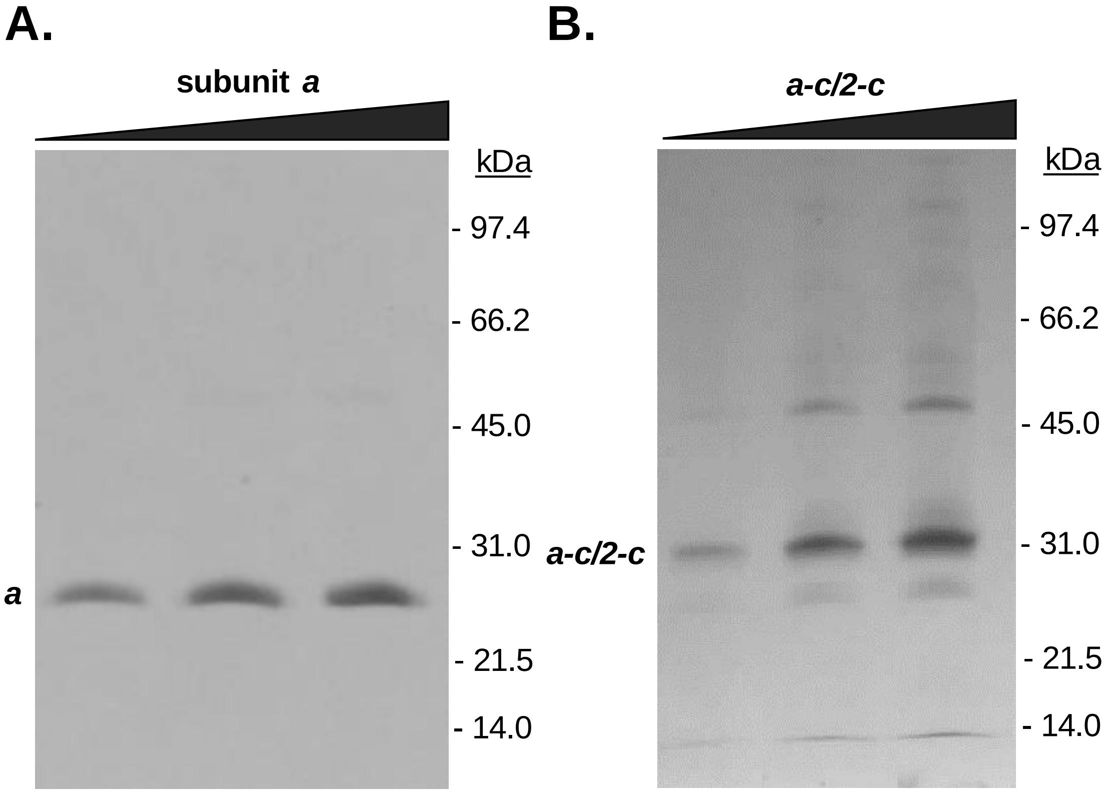

Purified subunit *a* and the *a-c/2-c* protein. Positions of the molecular weight standards (KDa) are indicated. (Coomassie stained gels with 1µg, 3 µg, and 5 µg purified subunit *a* (A) or the *a-c/2-c* protein (B). Note, that the electrophoretic mobility of subunit *a* and the *a-c/2-c* protein does not correspond to the actual molecular mass of the proteins (31 KDa and 45 KDa respectively). This has been often observed with highly hydrophobic proteins, including subunit *a* [37].

Fig. S2

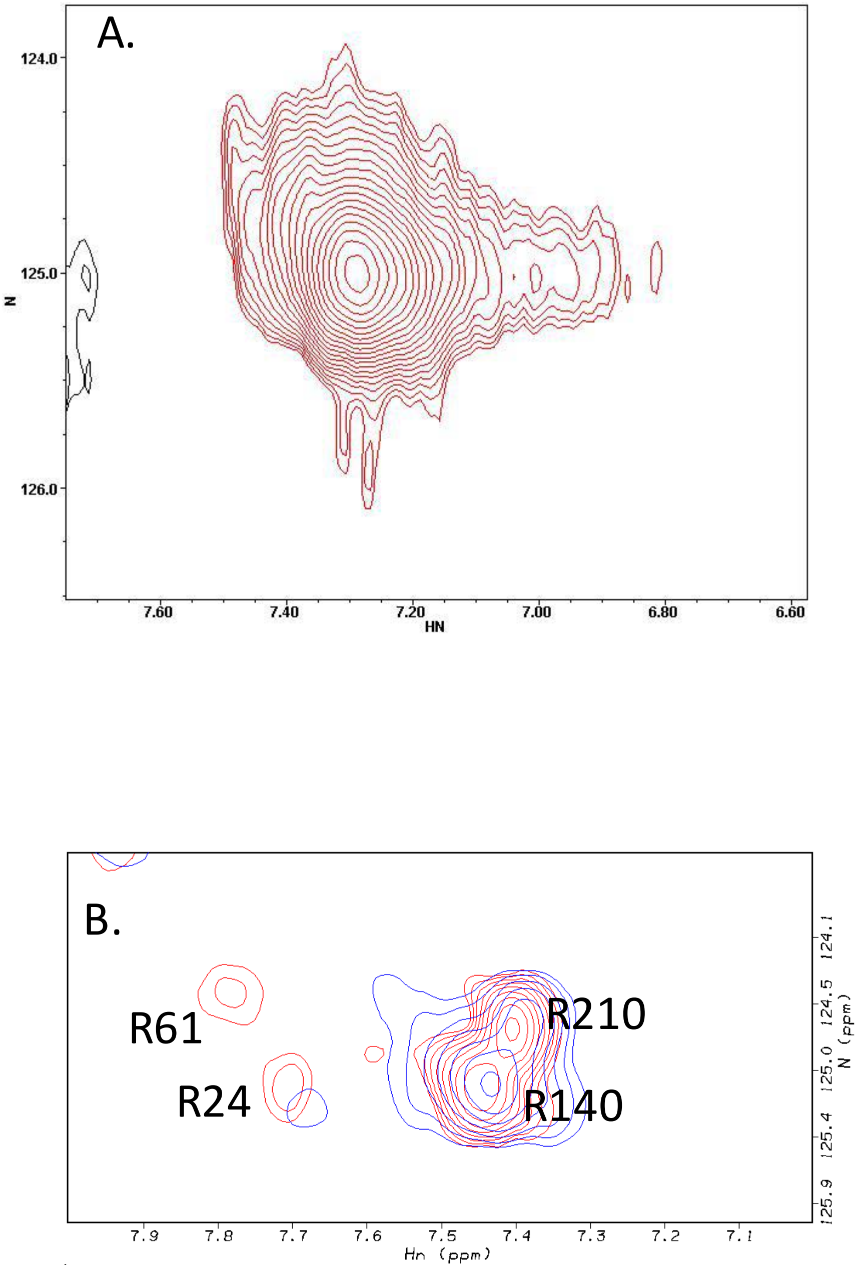

The arginine sidechain regions of the $^{1}H,^{15}N$-HSQC spectra the *a-c/2-c* protein. (A) The spectrum of the *a-c/2-c* protein with the wild type sequences of the subunit *a* and subunit *c* fragments; (B) Overlay of the spectra of the R283K/R292K/R373K/R382K variant of the *a-c/2-c* protein (*blue*) and of subunit *a* (*red*), with arginine side chain assignments shown.
